# Spatial muscle synergy-based network modeling and analysis of sit-to-stand transition with and without robot assistance

**DOI:** 10.3389/fbioe.2025.1543468

**Published:** 2025-05-08

**Authors:** Tianyi Wang, An Guo, Keisuke Shima, Yuko Ohno

**Affiliations:** ^1^ Institute for Multidisciplinary Sciences, Yokohama National University, Yokohama, Kanagawa, Japan; ^2^ Department of Mathematical Health Science, Graduate School of Medicine, Osaka University, Suita, Osaka, Japan; ^3^ Faculty of Environment and Information Science, Yokohama National University, Yokohama, Kanagawa, Japan

**Keywords:** assistance robot, human–robot interaction, muscle coordination, muscle network, sit-to-stand transition

## Abstract

The sit-to-stand (STS) transition is crucial for daily activities, and it is particularly challenging for those with physical disabilities. This paper investigates the dynamics of muscle synergy networks during the STS transition, comparing self-executed STS with robotic assistance. Six subjects participated in the study, performing STS with and without robotic assistance. Muscle coordination was assessed using electromyography data from the trunk, thigh, and shank muscles. Non-negative matrix factorization (NMF) was employed to extract muscle coordination patterns, revealing distinctions in the number of synergies between self- and robot-STS. Spatial muscle synergy analysis indicated significant differences between self- and robot-STS, emphasizing alterations in muscle activation patterns due to robotic assistance. Detailed muscle-level analysis highlighted specific muscles’ modulation, particularly in the shank, thigh, and trunk regions. Network analysis demonstrated variations in coordination network connectivity and stability between self- and robot-assisted STS. Centrality measures identified specific muscles, such as vastus lateralis, playing a crucial role in dynamic correlations within the muscle synergy network during STS. The findings suggest adaptability in human motor system responses to external assistance, with implications for refining robotic assistance strategies to align with natural movement patterns. Future research could involve a more diverse participant pool and explore upper-limb support.

## 1 Introduction

The sit-to-stand (STS) transition is a vital component of daily life, facilitating the shift from a seated to a standing position. Bohannon (2015) reports that, on average, individuals perform the STS transition 45 times daily, with this figure increasing to 71 for healthy older individuals. Recognized as the starting point for most activities of daily living (ADL), STS is acknowledged as one of the most challenging and mechanically demanding activities (Yamako et al., 2017). However, individuals facing physical disabilities and unable to execute the STS transition experience a diminished quality of life, necessitating assistance. Unfortunately, the escalating aging population and a shortage of caregivers pose challenges for meeting this substantial demand.

**FIGURE 1:**
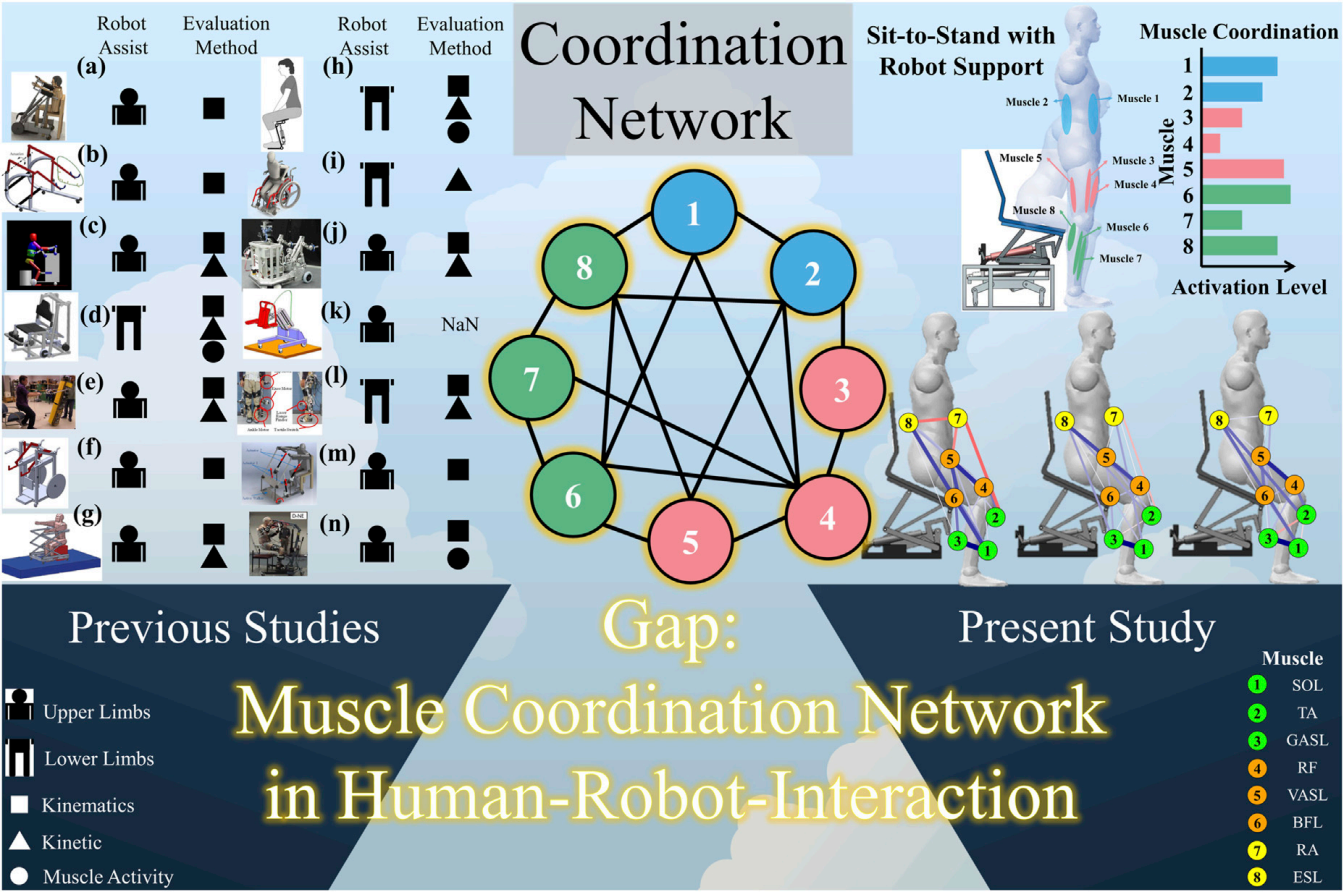
Related works about STS assistance robots, research gap, and the concept of this study. **(a)** Asker and Assal (2019). **(b)** Rea and Ottaviano (2018). **(c)** Mombaur and Ho Hoang (2017). **(d)** Matjačić et al. (2016). **(e)** Shomin et al. (2015). **(f)** Ottaviano et al. (2014). **(g)** Rea et al. (2013). **(h)** Zheng et al. (2019). **(i)** Shiraishi and Sankai (2018). **(j)** Geravand et al. (2017). **(k)** Rea and Ottaviano (2016). **(l)** Kiguchi and Yokomine (2015). **(m)** Asker et al. (2014). **(n)** Burnfield et al. (2013).

To address this challenge, diverse assistance robots have been developed. These robots vary in design and function (refer to Figure 1 for visual examples of STS assistance robots) and are primarily categorized into upper-limb support (Asker and Assal, 2019; Rea and Ottaviano, 2018; Mombaur and Ho Hoang, 2017; Geravand et al., 2017; Rea and Ottaviano, 2016; Shomin et al., 2015; Ottaviano et al., 2014; Asker et al., 2014; Rea et al., 2013; Burnfield et al., 2013) and lower-limb support (Zheng et al., 2019; Shiraishi and Sankai, 2018; Matjačić et al., 2016; Kiguchi and Yokomine, 2015). Various approaches have been explored in the development of robotic assistance for STS, with each addressing unique challenges and preferences. Notably, the assistance provided from the shoulder has been deemed effective for maximal support by service robots (Asker and Assal, 2019; Rea et al., 2013). Concurrently, trunk support has gained popularity as a prevalent method for aiding STS. In contrast, a distinct perspective is observed among robotics researchers and engineers who advocate for enabling individuals to use their own arm power to perform the STS task (Geravand et al., 2017; Shomin et al., 2015; Burnfield et al., 2013). This divergent approach underscores the diversity in the strategies employed to tackle the challenges of STS assistance.

Further attention has been directed toward supporting STS from the lower limbs, acknowledging the increased torque demand during STS compared to walking (Mak et al., 2003). The inadequacies of some upper-limb support robots, highlighted by their weight, bulkiness, and maneuverability issues, have prompted a shift toward lower-limb support (Zheng et al., 2019). This shift encourages user participation by allowing real-time regulation of torque outputs, fostering a more dynamic and user-engaged STS process. In response to these challenges, innovative robotic designs have emerged, including a wheel-chair-type robot supporting lower limbs (Shiraishi and Sankai, 2018) and a chair-type robot developed by Matjačić and colleagues for providing STS support services (Matjačić et al., 2016).

When a robot supports the STS transition, the interaction between the robot and the user becomes a crucial factor in determining the success of the assistance. Evaluation of STS assistance robots has thus become a prominent focus within the field of human–robot interaction. Approaches include assessing the center of mass calculated from a human model (Geravand et al., 2017; Rea and Ottaviano, 2016), applying the minimum jerk criterion of shoulder trajectory for replicating natural STS performance (Asker and Assal, 2019), trajectory verification through shoulder trajectory recording (Shomin et al., 2015; Rea and Ottaviano, 2018), and studying lower-limb activities, with a specific emphasis on knee angular displacement (Zheng et al., 2019).

In-depth investigations into various aspects of human motion, such as ankle, knee, hip, pelvis, and trunk displacement, have been conducted to provide a comprehensive understanding of STS dynamics (Matjačić et al., 2016). The study of knee torque (Zheng et al., 2019), vertical ground reaction force (Shiraishi and Sankai, 2018; Matjačić et al., 2016), and electromyography (EMG) analyses focusing on muscles such as vastus lateralis, vastus medialis, and rectus femoris (Zheng et al., 2019) contributes valuable insights into the intricate dynamics of performing STS tasks when assisted by a robot. Similar studies extending to multiple lower-limb muscles, including the tibialis anterior, soleus, gastrocnemius, quadriceps, and hamstrings, have enriched our knowledge of muscle activities during STS (Matjačić et al., 2016).

Additionally, research efforts have extended to exploring human intention during STS when utilizing wearable robots, employing 8-channel EMG testing, (Kiguchi and Yokomine, 2015). Building upon these endeavors, our previous studies have evaluated STS assistance robots from diverse perspectives, including motion coordination, center of mass (CoM), center of pressure (CoP), and muscle synergy (Wang et al., 2017; Wang et al., 2018a; Wang et al., 2018b, Wang et al., 2021). These investigations collectively share the common theme of assessing robot assistance from the standpoints of coordination and synergy.

Despite these advancements, a critical gap remains in the understanding of the network of muscle synergy during STS when assisted by healthcare robots. Given the interdependence and varied synergies among STS-related muscles, this study seeks to unravel the intricacies of the muscle synergy-based network during STS transitions performed both with and without robot assistance. The findings from this study are anticipated to offer profound insights into the realm of human–robot interaction, specifically for assistance robots in the context of STS.

The contributions of the present study are as follows:

• Network-based muscle synergy characterization in robotic STS: while existing studies have focused on isolated muscle activation patterns during STS, this work pioneers the integration of network science with muscle synergy analysis. By employing non-negative matrix factorization and graph-theoretical metrics, we systematically reveal how robotic assistance reconfigures the topological structure and dynamic correlations within the muscle synergy network. This approach provides a novel framework to quantify the adaptability of neuromuscular control strategies under external interventions.

• Spatial modulation of muscle coordination: beyond conventional comparisons of synergy counts, this study identifies region-specific modulation of muscle coordination (shank, thigh, and trunk) induced by robotic assistance. The observed hierarchical reorganization of activation patterns—particularly the enhanced centrality of the vastus lateralis in robot-assisted STS—uncovers compensatory mechanics where humans prioritize proximal joint stabilization during assisted motion.

• Design principles for adaptive robotic assistance: the study establishes quantitative links between network-level synergy properties (connectivity stability and node centrality) and human–robot interaction during STS transitions. By demonstrating that robotic assistance alters not just muscle activation magnitude but also inter-muscular coordination topology, our results propose a paradigm shift in assistive robot design: rather than merely reducing biomechanical loads, optimal assistance should preserve or enhance the natural network properties of neuromuscular coordination. This insight directly informs the development of human-in-the-loop control strategies for personalized rehabilitation robotics.

## 2 Methods

### 2.1 Subjects and experiment

Six healthy male subjects (age: 25.8 ± 2.5 y.o., height: 1.78 ± 0.02 m, BMI: 22.65 ± 3.14 ${kg/m}^{2}$ ) volunteered to join in this study. None of the subjects reported any lower-limb pathology, neurological disease, low back pain, or use of medications that could potentially influence their motor abilities. Approval for this study was obtained from the Ethics Committee of the Division of Health Science of the Graduate School of Medicine, Osaka University ((No. 305, 20140821).

**FIGURE 2:**
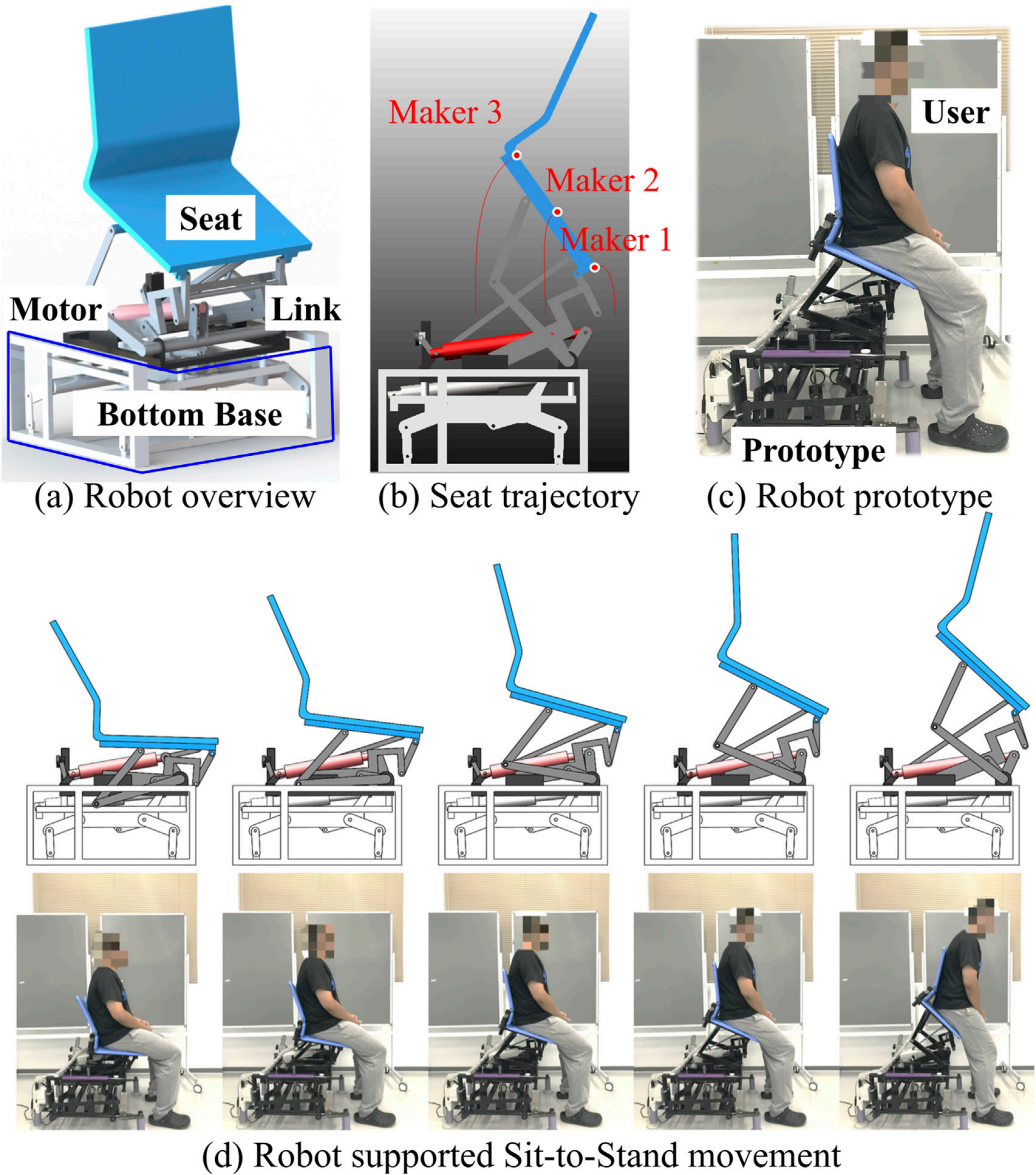
Overview of the STS support robot. **(a)** Robot overview. **(b)** Robot seat trajectories. **(c)** Robot prototype. **(d)** Robot-assisted sit-to-stand transition.

**FIGURE 3:**
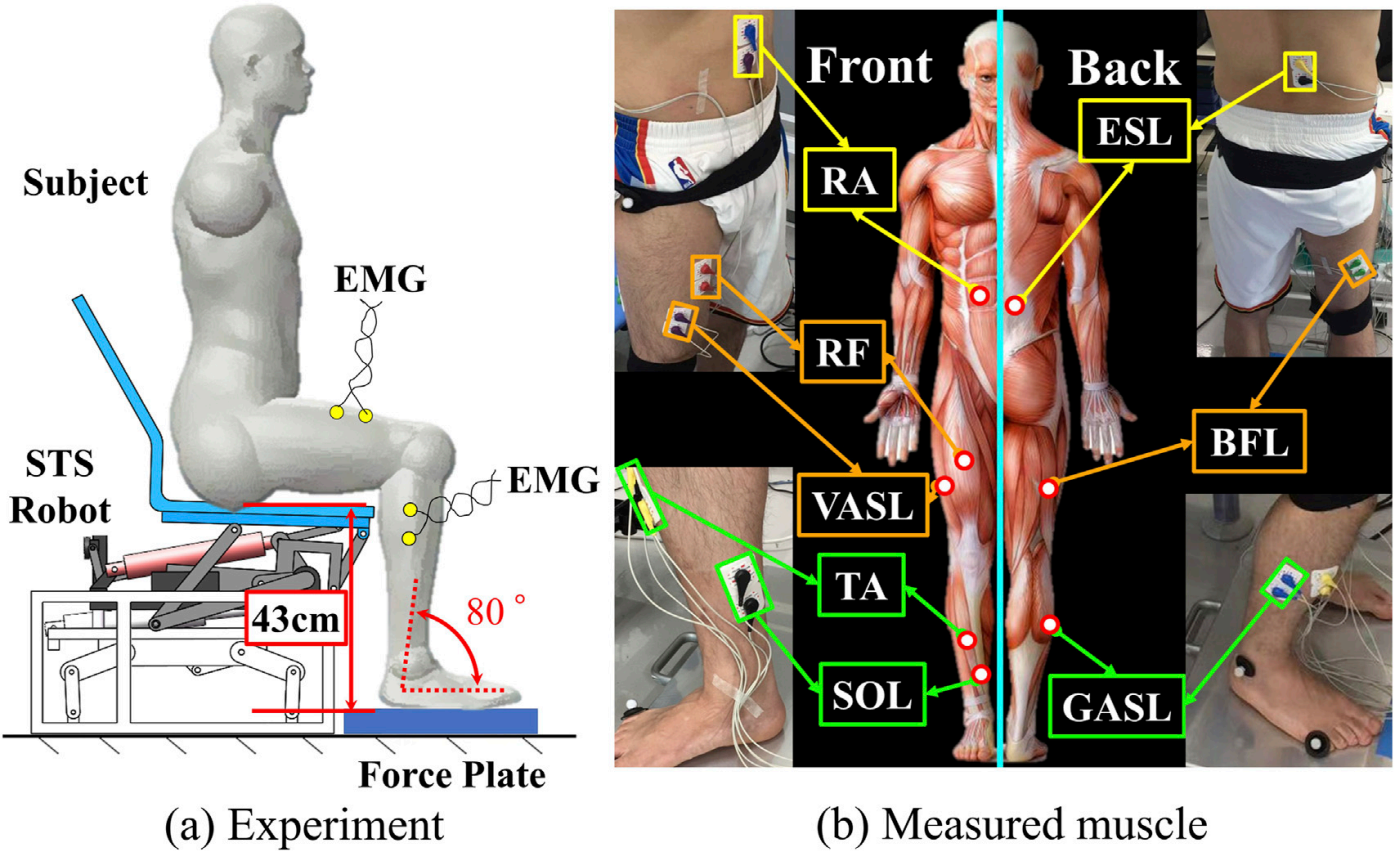
Experiment overview. **(a)** Experiment environment and **(b)** measured muscle.

Figure 2a provides an overview of the STS support robot, as developed in our prior work (Jeong et al., 2019). This robot is composed of four main parts: a seat, four-bar links, a motor, and a bottom base. The STS motion is facilitated through the vertical and rotational movements of the robot seat. Trajectories of three markers on the robot seat are visualized in Figure 2b. The prototype of the robot and an illustrative example of STS supported by the robot are presented in Figures 2c, d, respectively.Figure 3a illustrates the experimental environment, while Figure (b) highlights the muscles that were measured. To assess and compare muscle coordination during STS movements with and without robot support, two experimental conditions were established. Participants were instructed to sit on the robot having a seat height of 43 cm, maintaining an angle of 80° between the foot and crus. Each participant then performed five STSs without robotic assistance (self-STS) and five with robotic assistance (robot-STS). This experimental design aimed to capture and analyze the nuances in muscle coordination under both conditions.

In both experimental conditions, eight muscular activities were recorded, encompassing two from the trunk (upper rectus abdominis (RA) and erector spinae longissimus (ESL)), three from the thigh (rectus femoris (RF), vastus lateralis (VASL), and biceps femoris long head (BFL)), and three from the shank (tibialis anterior (TA), soleus (SOL), and gastrocnemius lateral head (GASL)). A biological amplifier (PL3516 PowerLab ADInstruments) with a sampling frequency of 1,000 Hz was employed for data acquisition (Wang et al., 2021). Electrode placement adhered to the recommendations outlined by the surface electromyography for the non-invasive assessment of muscles (SENIAM) (Hermens et al., 1999). Raw EMG data underwent pre-processing, including an 80–200 Hz bandpass filter with a zero-leg fourth-order Butterworth filter, rectification, and a 0.2 s moving average. Singular spectrum analysis (SSA) was employed to eliminate the electrocardiographic signal at RA and ESL. For each subject, EMG data were normalized to the maximum amplitude observed during ten trials (five for self-STS and five for robot-STS) to eliminate the possible effect of bias due to subject-independent factors such as height and weight. Synchronization between muscle activities and STS movements was achieved using a force platform beneath the subject’s feet (TF-4060, Tec Gihan Co.) with a sampling frequency of 1,200 Hz. Time was normalized to 100%.

### 2.2 Muscle coordination

In the muscle coordination model, muscle activation can be represented as a linear summation of spatiotemporal patterns, expressed as follows:

$$M\approx \left(\begin{array}{cccc} W_{1} & W_{2} & \cdots & W_{n} \end{array}\right)\left(\begin{array}{c} C_{1}\left(t\right)\\ C_{2}\left(t\right)\\ \vdots \\ C_{n}\left(t\right) \end{array}\right),$$ (1)

where M represents the muscle activity; W is the relative activation level (spatial pattern), namely, the relative activity ratio of multiple muscles; C represents the change in muscle activation over time (temporal pattern), which denotes the activation profiles of each muscle coordination; i(i=1,2,…,n) is the number of muscles; and t represents the time.

**FIGURE 4:**
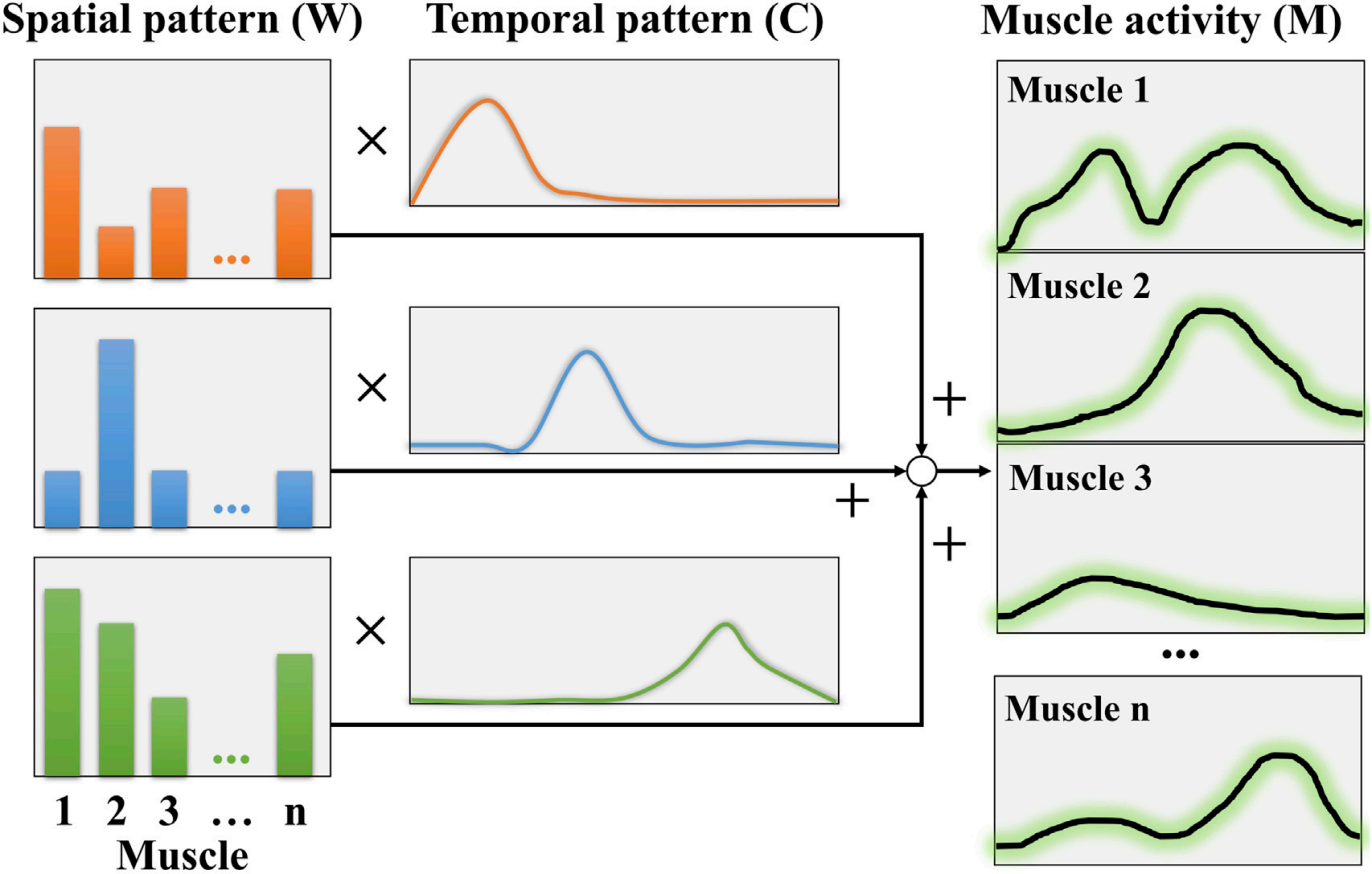
NMF concept for muscle coordination analysis.

Non-negative matrix factorization (NMF) was employed for extracting muscle coordination (Hanawa et al., 2017). Figure 4 shows an example of muscle coordination analysis using NMF. The fundamental concept of NMF is to establish an optimization process to determine the matrices W and C by minimizing the reconstruction error e=M−WC :

$$\underset{W\geq 0,C\geq 0}{\mathrm{argmin}}\|M-WC\|.$$ (2)

The variance accounted for (VAF) is subsequently calculated to determine the number of synergies, as follows:

$$VAF=1-\frac{\sum_{i=1}^{n}\sum_{t=t_{\mathrm{start}}}^{t_{\mathrm{end}}}{\left(e_{i,t}\right)}^{2}}{\sum_{i=1}^{n}\sum_{t=t_{\mathrm{start}}}^{t_{\mathrm{end}}}{\left(M_{i,t}\right)}^{2}},$$ (3)

where i(i=1,2,…,n) denotes the number of muscles and t represents time. The determination of the number of synergies during STS is based on a significant difference criterion (Wang et al., 2021). Once the number of synergies is determined, the spatial patterns of muscle coordination (muscle activation level W ) in each STS condition under different synergies are utilized to construct and analyze the network.

### 2.3 Spatial coordination network

In this study, we utilized the graphical model to study the relation among different muscle groups involved in coordination. In the graphical model G=(V,E) , where V represents the nodes; in our study, nodes are the spatial pattern of muscle coordination, and E is the edge that represents the connections between two nodes. Make *X* = ${\left\{x_{i}\right\}}_{i=1,\ldots q}$ , $x_{i}\in \chi$ , where x is the muscle coordination; then, the two muscles $x_{i}$ and $x_{j}$ are connected with an edge only if they are conditionally dependent, given the other nodes in the network (i,j)∈E (Yuan and Lin, 2007). By modeling GM for spatial muscle coordination, we can acquire a meaningful interpretation of the network as a roadmap containing information regarding which muscles are directly associated under a given human–robot interaction situation. GM can be divided into two main parts, namely, the Ising graphical model for binary data and the Gaussian graphical model (GGM) for continuous variables. In this study, we mainly focused on the GGM.

The GGM is based on the multivariate Gaussian distribution holding the following density function:

$$p\left(x|\mu ,\Sigma \right)=\frac{1}{{\left(2\pi \right)}^{n/2}|\Sigma {|}^{1/2}}exp\left\{-\frac{1}{2}{\left(x-\mu \right)}^{⊤}\Sigma^{-1}\left(x-\mu \right)\right\},$$ (4)

where, without loss of generality, we assume μ=0 and define Θ as the inverse of the covariance matrix; Θ is also called the precision matrix. Let μ=0 , $\Theta =\Sigma^{-1}$ ; then, Equation 4 can be written as follows:

$$p\left(x|\mu =0,\Theta \right)=\frac{|\Theta {|}^{1/2}}{{\left(2\pi \right)}^{n/2}}exp\left\{-\frac{1}{2}x^{⊤}\Theta x\right\},$$ (5)

where if $\theta_{ij}=0$ , there is no connection between node i and j ; that is, $\theta_{ij}=0\Leftrightarrow x_{i}⊥x_{j}|x\backslash \left\{x_{i},x_{j}\right\}\Leftrightarrow \left(i,j\right)\notin E$ . In the GGM, by estimating the inverse of the covariance matrix Θ , we can evaluate the network, known as covariance selection (Dempster, 1972). The most popular approach for solving this question is to use log likelihood using the extended Bayesian information criterion graphical least absolute shrinkage and selection operator (EBICglasso) (Foygel and Drton, 2010).

The main principle of EBICglasso mainly contains two parts. First, glasso seeks to directly maximize the log likelihood by using a regularizer (penalty function). Second, EBIC is used to decide the tuning parameter. The glasso algorithm takes the covariance matrix as the input and outputs the sparse precision matrix, here Θ, which maximizes the log likelihood, as following:

$$\hat{\Theta }=\underset{\Theta }{\mathrm{arg min}}\left\{ln|\Theta |-tr\left(S\Theta \right)-\rho \|\Theta {\|}_{1}\right\},$$ (6)

where ln|Θ|−tr(SΘ) is the log-likelihood, and $\rho \|\Theta {\|}_{1}$ is the regularizer. Then, the tuning parameter is tested using the following EBIC:

$${BIC}_{\lambda }\left(E\right)=-2ln\left(\hat{\Theta }\left(E\right)\right)+|E|log\;n+4|E|\lambda log\;p,$$ (7)

$$E_{0}=\underset{E\in \varepsilon }{\mathrm{arg min}}{BIC}_{\lambda }\left(E\right),$$ (8)

where E is the edge of the graph, and $ln\left(\hat{\Theta }\left(E\right)\right)$ represents the maximized log-likelihood function. In this study, the tuning parameter λ was set at 0.5. The EBIC with parameter λ 0.5 selects the smallest true model $E_{0}$ when applied to any subset of all decomposable models ε containing $E_{0}$ .

In this study, centrality measures including degree, betweenness, closeness, and strength were also employed to evaluate the network (Saxena and Iyengar, 2020). The degree indicates the number of connections incident to the node of interest, betweenness measures the importance of the node in the average pathway between other pairs of nodes, closeness quantifies the relationship to all other nodes, and strength indicates how strong a node is directly connected to other nodes, highlighting the node’s importance.

For a given graph G(V,E) , V is the node, E is the edge, n is the total number of nodes, m is the total number of edges, $k_{u}$ is the total number of neighbors of the node u , and d(u,v) is the distance between the nodes u and v .

Then, the degree centrality $C_{D}\left(u\right)$ is defined as follows:

$$C_{D}\left(u\right)=\frac{k_{u}}{n-1}.$$ (9)

The betweenness centrality $C_{B}\left(u\right)$ is defined as follows:

$$C_{B}\left(u\right)=\frac{\Sigma_{s\neq u\neq t}\frac{\partial_{st}\left(u\right)}{\partial_{st}}}{\left(n-1\right)\left(n-2\right)/2},$$ (10)

where $\partial_{st}\left(u\right)$ is the number of the shortest path between the nodes s and t, with node u acting as an intermediate node in this shortest path.

The closeness centrality $C_{C}\left(u\right)$ is defined as follows:

$$C_{C}\left(u\right)=\frac{n-1}{\Sigma_{\forall v,v\neq u}d\left(u,v\right)}.$$ (11)

The strength S(u) is defined as follows:

$$S\left(u\right)=\Sigma_{v}w_{uv}.$$ (12)

To assess the stability of edges and centrality, 200 non-parametric bootstraps were conducted with a significance level α = 0.01 ( $\alpha =2/N_{b}$ , $N_{b}$ is the bootstrap). In addition, network similarity was evaluated using the DeltaCon comparison for two pairs (Koutra et al., 2013).

### 2.4 Statistical analysis

The VAFs and all spatial patterns of muscle synergy were analyzed using JASP (version 0.18.1.0, Netherlands). The Shapiro–Wilk test was employed as the normality test, and Levene’s test was used to confirm the equality of variances. Based on the results of appropriate tests, non-parametric one-way and two-way ANOVA (Kruskal–Wallis test) were used to ascertain significant differences in VAFs and muscle synergy, respectively. Dunn’s *post hoc* comparison was performed if a significant difference was observed. The significance level was set at 5%. Network modeling, analysis of centrality, and stability assessments were conducted using JASP network analysis. Network comparison was performed using MATLAB (version R2023a).

## 3 Results

### 3.1 Spatial muscle coordination

**FIGURE 5:**
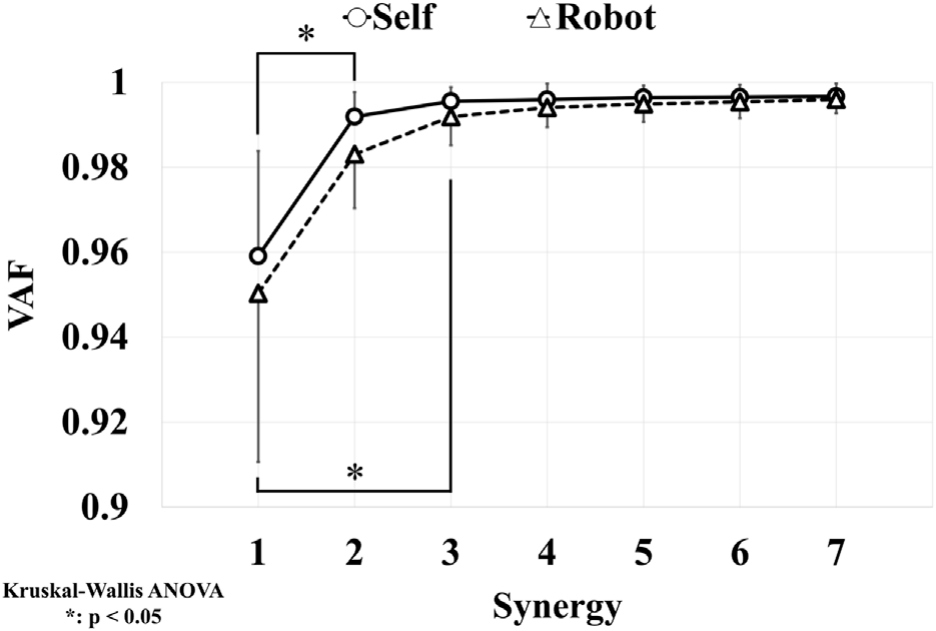
Results of VAFs. The number of synergy is determined through statistically significant differences.

Figure 5 illustrates the results of the VAF. VAFs for all seven analyzed synergies in both self- and robot-STS were higher than 0.9. According to the Kruskal–Wallis test, for self-STS, there was a significant difference between the first and second synergy (p < 0.05); for robot-STS, there was a significant difference between the first and third synergy (p < 0.05). Thus, the number of synergies for self- and robot-STS was determined to be 2 and 3, respectively.

**FIGURE 6:**
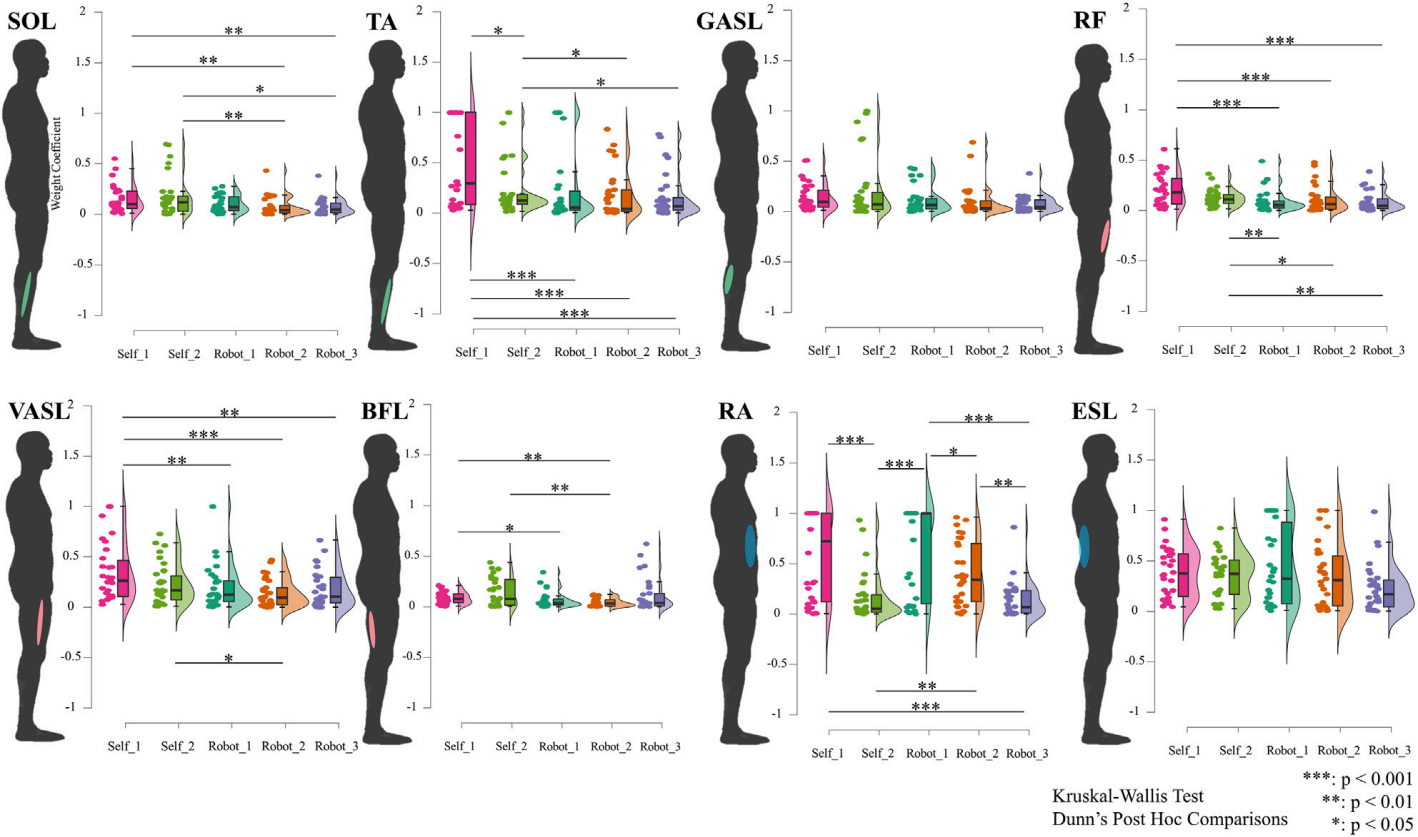
Results of spatial muscle synergy. Data are ordered by each muscle (shown at the human model), plotted using raincloud plots. The horizontal axis indicates different STS synergies, and the vertical axis represents the weight coefficient of spatial muscle synergy. All spatial muscle synergies are tested using the Kruskal–Wallis ANOVA test. Dunn’s *post hoc* comparisons are carried out when significant differences are reported.

Figure 6 presents the results of spatial muscle synergy. According to the Dunn’s *post hoc* comparisons, the spatial muscle synergy exhibited a significant difference when the STS was supported by the robot compared to self-STS, except for GASL and ESL. In the shank muscles, for the SOL muscle, the weight coefficients for robot-STS synergies 2 and 3 were smaller than those for self-STS (robot 2: 0.074 ± 0.094; robot 3: 0.074 ± 0.080; self 1: 0.149 ± 0.135; self 2: 0.176 ± 0.202, and all p < 0.05). There was no significant difference between self-STS and neither between robot-STS synergies 2 and 3 compared to synergy 1. For the TA muscle, self-STS synergy 1 had the highest weight coefficient compared to other synergies (0.511 ± 0.436). Self-STS synergy 2 had a higher coefficient than robot-STS synergies 2 and 3 (self 2: 0.219 ± 0.249; robot 2: 0.183 ± 0.240; robot 3: 0.164 ± 0.223, and all p < 0.05). There was no significant difference between self-STS, and neither between robot-STS synergies 2 and 3 compared to synergy 1. In the muscles at the thigh, for the RF muscle, although there was no significant difference between self-STS, synergies in self-STS were significantly higher than all robot-STS synergies (self 1: 0.202 ± 0.156; self 2: 0.125 ± 0.083; robot 1: 0.085 ± 0.106; robot 2: 0.105 ± 0.130; robot 3: 0.083 ± 0.095, and all p < 0.05). There was no difference among the three robot-STS synergies. Spatial muscle synergy 1 under self-STS at the VASL muscle was significantly higher than all robot-STS synergies (self 1: 0.346 ± 0.288; robot 1: 0.190 ± 0.214; robot 2: 0.140 ± 0.136; robot 3: 0.180 ± 0.183, and all p < 0.01). Self-STS synergy 2 (0.234 ± 0.202) showed a higher value than robot-STS synergy 2. There was no difference among the three robot-STS synergies. For the BFL muscle, robot-STS synergy 2 was significantly lower than self-STS synergies (robot 2: 0.041 ± 0.040; self 1: 0.086 ± 0.059; self 2: 0.138 ± 0.139, and all p < 0.01). Robot-STS synergy 1 (0.065 ± 0.080) was smaller than self-STS synergy 1. There was no difference among the three robot-STS synergies. For the RA muscle at the trunk, synergy 1 under self-STS was higher than synergy 2 and robot-STS synergy 2 (self 1: 0.577 ± 0.436; self 2: 0.166 ± 0.239; robot 3: 0.139 ± 0.179, and p < 0.001). Synergy 2 under self-STS was significantly smaller than robot-STS synergies 1 and 2 (robot 1: 0.642 ± 0.434; robot 2: 0.393 ± 0.315; and p < 0.01). Robot synergy 1 had the highest value in the robot-STS, and robot synergy 2 had higher value than robot synergy 3.

### 3.2 Muscle coordination network

Table 1 summarizes the estimated networks. The network for self-STS synergy 2 had the most non-zero edges among all networks, and the sparsity was smaller than that of other networks. Spatial muscle synergy networks did not dramatically change for the robot-STS.

**TABLE 1:** Summary of the network.

Network	Nodes	Non-zero edges
Self-synergy 1	8	17/28
Self-synergy 2	8	20/28
Robot synergy 1	8	18/28
Robot synergy 2	8	18/28
Robot synergy 3	8	19/28

**FIGURE 7:**
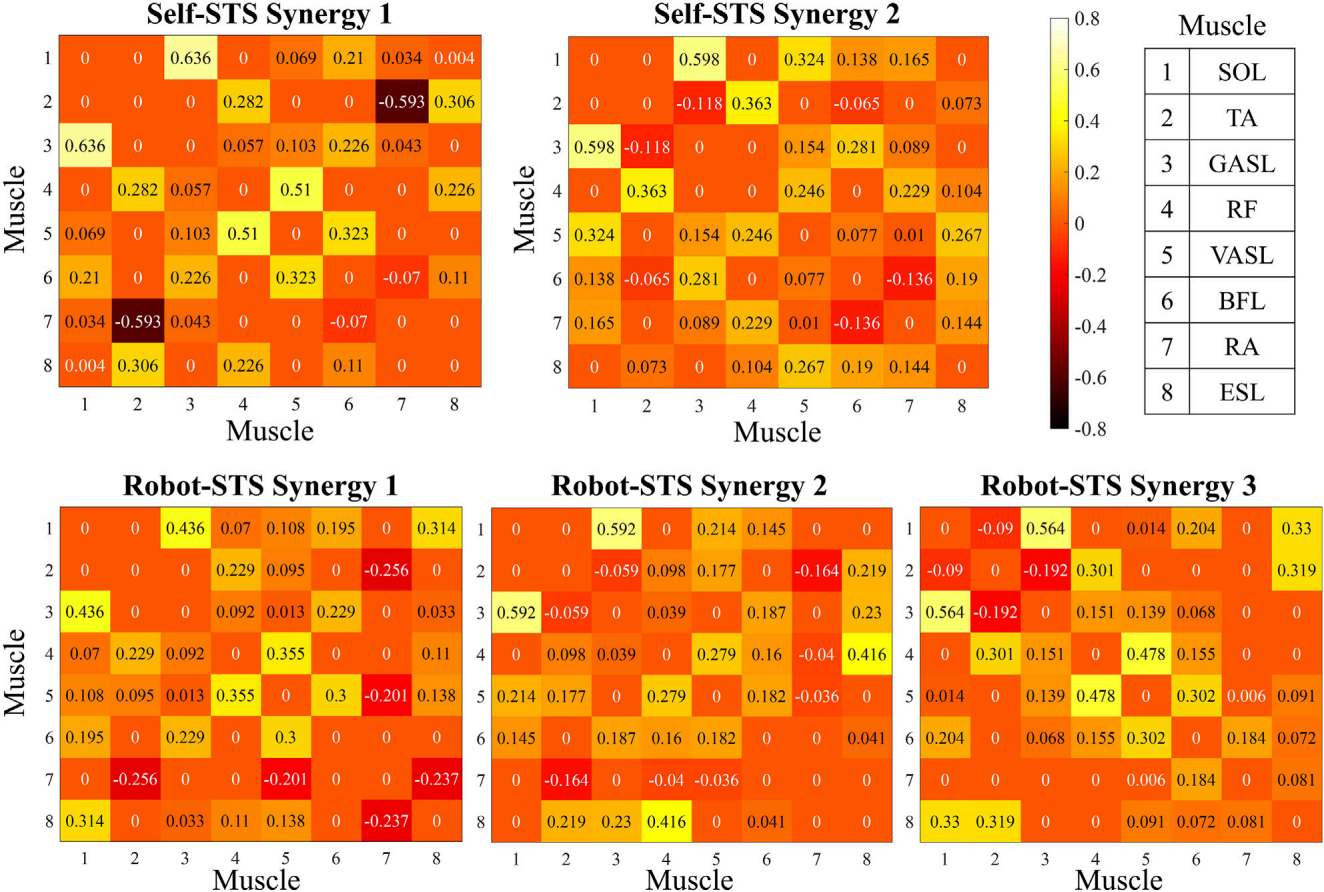
Results of the node weight matrix. Node weight is plotted with a heat map.

**FIGURE 8:**
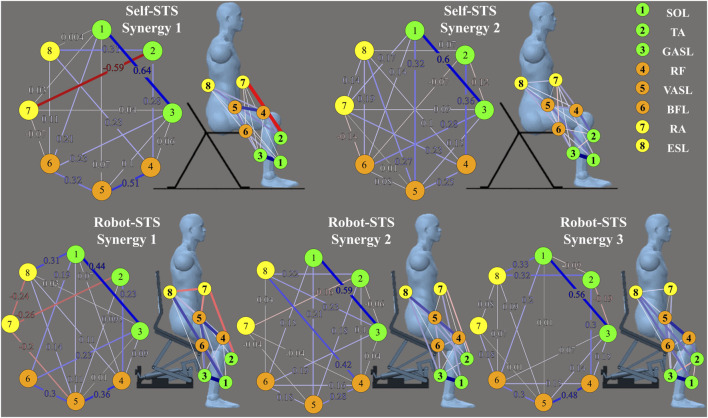
Results of graphical network structures. The graphical network at the left part is plotted according to the results shown in Figure 7, and the graphical network at the right part indicates the muscle coordination network under different experimental conditions with the muscle position.

Heat maps in Figure 7 illustrate the results of the weight matrix for each muscle synergy network. For all synergies under both self- and robot-STSs, the network weight between muscles 1 and 3 (SOL and GASL) had the highest values, i.e., self 1: 0.636, self 2: 0.598, robot 1: 0.436, robot 2: 0.592, and robot 3: 0.564, indicating that nodes 1 and 3 hold the strongest positive correlation. The TA muscle at self-STS synergy 1 and all robot-STS synergies showed the highest negative correlation between RA (self 1: –0.593, robot 1: –0.256, and robot 2: –0.164) and GASL (robot 3: –0.192). In self-STS synergy 2, the highest negative correlation was between BFL and RA (−0.136). Then, the graphical network can be plotted using the node weight. In the graphical network, each node represents one muscle, and the line between two nodes indicates the edge, namely, the node weight. Blue lines indicate a positive correlation, and red lines indicate a negative correlation. Thin lines represent a weak association, and thick lines represent a strong association. The spatial muscle synergy networks are plotted in Figure 8.

**FIGURE 9:**
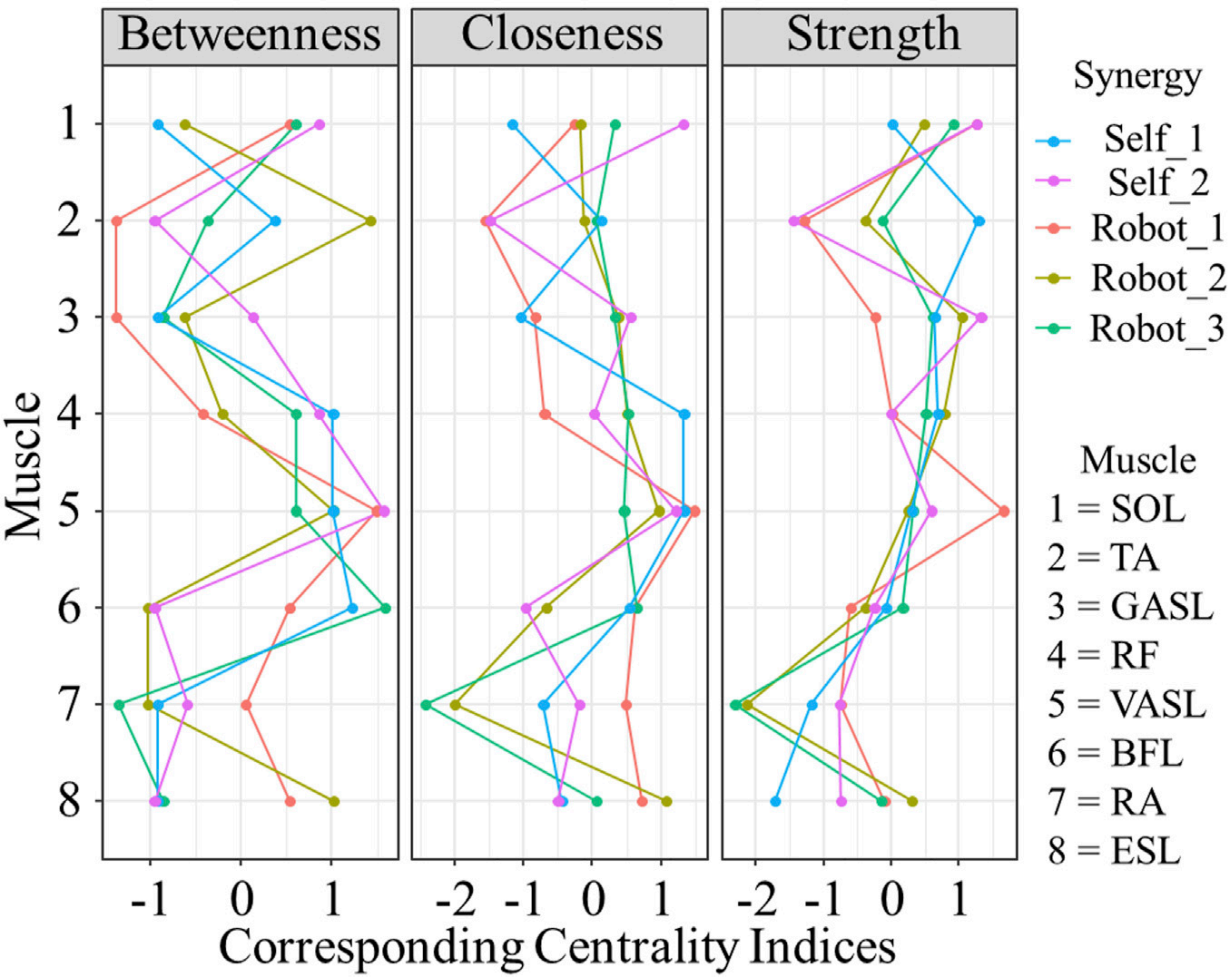
Results of corresponding centrality indices. Data are shown as standardized z -scores.

Results of centrality of networks are plotted in Figure 9. All measured muscle synergies showed different centrality both for self- and robot-STSs. Muscle No. 5, VASL, showed higher betweenness, closeness, and strength on average (all values > 0), indicating that VASL played an essential role in the muscle synergy dynamic correlation. Specifically, the VASL muscle was important in the average pathway, shared the most shortest pathway from other nodes, and had a strong direct connection to other nodes.

**FIGURE 10:**
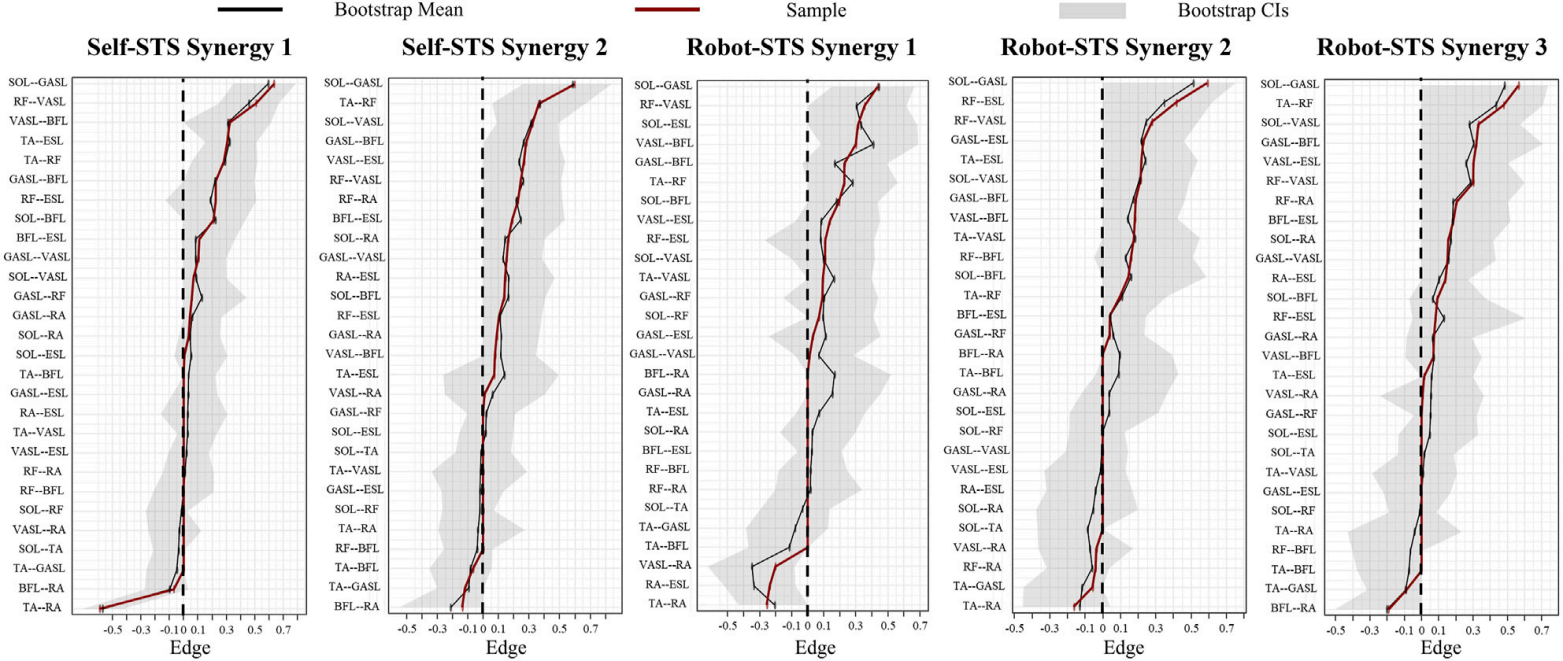
Results of bootstrapped confidence intervals of edge weights. Black lines represent the mean value after bootstrap, red lines indicate the sample value, and gray areas are the bootstrap CIs. Data are ordered from the edge with the highest edge-weight to the edge with the lowest edge-weight.

Figure 10 illustrates the results of edge stability. Sizable bootstrapped confidence intervals (CIs) indicated that many edge weights did not significantly differ from other nodes. Compared to self-STS synergy 2 and all robot-STS synergies, the bootstrap CI in self-STS synergy 1 was narrower, indicating higher edge-weight accuracy in self-STS synergy 1. Edge weights between SOL and GASL muscles were the strongest in all synergy networks. From the top five strongest edge-weights, self-STS synergy 1 had stronger edge stability in the thigh (RF-BFL and VASL-BFL), while self-STS synergy 2 had stronger edge stability between the shank and thigh (TA-RF, SOL-VASL, and GASL-BFL). In the robot-STS synergy 1, strong edge-weight stability was found more in the lower limbs than in the trunk. In the robot-STS synergy 2, edge weights correlated to the trunk (ESL muscle) were stronger than that of other nodes (RF-ESL, GAL-ESL, and TA-ESL). In the robot-STS synergy 3, strong edge-weight stability was found at the muscles at the shank (SOL-GASL, TA-RF, SOL-VASL, and GASL-BFL).

**FIGURE 11:**
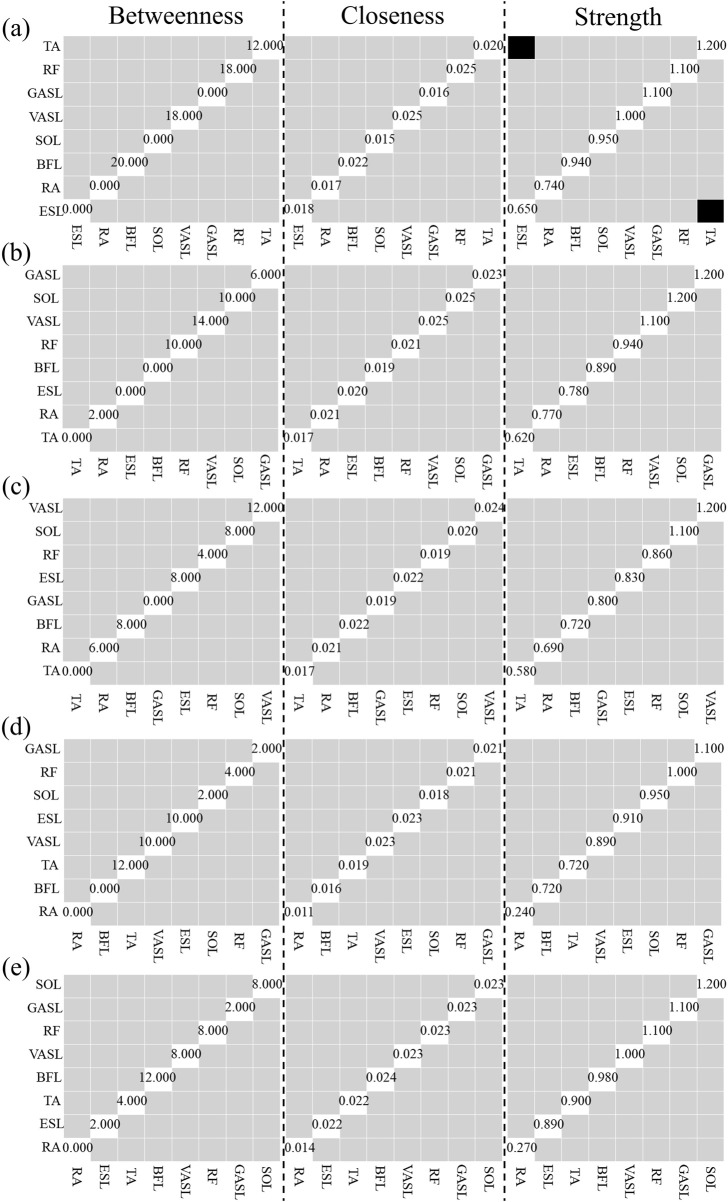
Results of Centrality Stability with bootstrap difference tests (*α* = 0.01). **(a)** Self-STS Synergy 1, **(b)** Self-STS Synergy 2, **(c)** Robot-STS Synergy 1, **(d)** Robot-STS Synergy 2, **(e)** Robot-STS Synergy 3. First column is betweenness, second column is closeness, third column is degree. Black boxes indicate nodes or edges that significantly differ from one other, gray boxes represent nodes or edges that do not significantly differ from one other, white boxes show the value of strength. Data are ordered from the muscle with the highest degree strength to it with the lowest degree strength.

Figure 11 illustrates the results of centrality stability. Most nodes’ betweenness and closeness for both self- and robot-STS synergy did not show a significant difference from those of other nodes. The TA muscle, with the strongest node strength, is significantly larger than ESL.

Table 2 summarizes the network similarity results with two pairs of comparison. As can be observed from Figure 8, no obvious difference was observed in all networks; however, self-STS synergy 2 and robot-STS synergy 1 showed the highest similarity value, indicating that these two networks were the most different compared to the other two pairs of comparison.

**TABLE 2:** Network similarity.

A	B	C	D	sim (A, B)–sim (C,D)
Self 1	Self 2	Self 1	Robot 1	0.0114
Self 1	Self 2	Self 1	Robot 2	0.0154
Self 1	Self 2	Self 1	Robot 3	0.0023
Self 1	Self 2	Self 2	Robot 1	0.0213
Self 1	Self 2	Self 2	Robot 2	0.0123
Self 1	Self 2	Self 2	Robot 3	−0.0018

Self-synergy 2 exhibits more connections and lower sparsity, suggesting stronger node communication. On the other hand, there is not much difference between robot synergies, indicating that the synergy connections did not change significantly during robot use. However, they are slightly less sparse than self-synergy 2, possibly due to the strategy of robot assistance. In summary, the network analysis shows an increase in the edge density and a decrease in the sparsity. Higher sparsity indicates weaker node communication.

## 4 Discussion

The study delves into the intricate dynamics of muscle synergy networks during the STS transition, comparing self-executed STS with robotic assistance. The primary aim is to contribute insights into the human–robot interaction domain, specifically within the context of STS assistance. The results of this study shed light on key aspects of muscle coordination, spatial synergy, and network properties during STS under different conditions.

The examination of VAF in both self- and robot-assisted STS revealed high VAF values for all seven synergies, signifying effective coordination of muscle activities in both scenarios. Notably, the determination of the number of synergies revealed distinctions between self-STS and robot-STS, with two synergies identified for self-executed STS and three for robot-assisted STS. This finding implies nuanced variations in muscle coordination strategies when individuals perform the STS task with and without robotic support.

Spatial muscle synergy analysis further underscored the impact of robotic assistance on muscle coordination. Significant differences in spatial muscle synergy between self-STS and robot-STS conditions were observed, indicating alterations in muscle activation patterns induced by the assistance provided by the robotic system. These findings contribute valuable insights into the adaptability and modulation of muscle coordination in response to external assistance during the STS transition.

Detailed analysis at the muscle level provided a comprehensive understanding of the impact of robotic assistance on specific muscle groups. At the shank muscles (SOL and TA), differences in weight coefficients between self-STS and robot-STS synergies underscored the modulatory effect of robotic assistance on lower-limb muscles. Similarly, at the thigh muscles (RF, VASL, and BFL), the study revealed significant differences in synergy values, emphasizing the intricate adjustments in muscle activation induced by robot assistance. The examination of trunk muscles (RA and ESL) further highlighted the differential impact of robotic assistance on muscle synergies. Synergy 1 under self-STS exhibited higher values than synergy 2 and robot-STS synergy 3 in the RA muscle, emphasizing the role of robotic assistance in altering trunk muscle activation patterns.

The network analysis provided a holistic view of the muscle coordination dynamics during STS. The construction of muscle coordination networks revealed variations in the number of non-zero edges, with self-STS synergy 2 exhibiting the most extensive connectivity. The analysis of edge stability indicated differences in the robustness of edge weights, emphasizing the distinct stability profiles of self-executed and robot-assisted STS synergies. Centrality measures further highlighted the importance of specific muscles, with VASL demonstrating higher betweenness, closeness, and strength on average. This suggests the critical role of VASL in facilitating dynamic correlations within the muscle coordination network during the STS transition.

The findings of this study have implications for the design and implementation of robotic assistance in STS tasks. The observed variations in muscle coordination patterns and network dynamics underline the adaptability of the human motor system to external assistance. Understanding these adaptations is crucial for refining robotic assistance strategies to align with natural human movement patterns. The identification of specific muscles, such as VASL, as central nodes in the synergy network highlights potential targets for enhancing the effectiveness of robotic assistance. Tailoring assistance strategies to leverage the centrality of key muscles could lead to more intuitive and user-friendly robotic systems.

While this study provides valuable insights, certain limitations should be acknowledged. The sample size is relatively small, and the study focused on healthy male subjects. Future research could explore a larger and more diverse participant pool, including individuals with varying levels of mobility and potential users of robotic assistance. Janssen et al. identified three primary factors influencing STS performance: subject-related factors (e.g., age and medical condition), chair-related factors (e.g., chair with or without armrest), and strategy-related factors (e.g., speed and arm use) (Janssen et al., 2002). In this study, all participants performed the STS transition at their self-selected comfortable speed for both self-STS and robot-STS conditions. However, to the best of our knowledge, no studies have specifically investigated whether variations in STS speed influence the muscle coordination network. Understanding the impact of different STS speeds on muscle coordination patterns presents an interesting avenue for future research. Additionally, the study primarily focused on lower-limb robotic assistance during STS. Exploring the nuances of upper-limb support and diverse robotic designs could contribute to a more comprehensive understanding of human–robot interaction in STS tasks.

## 5 Conclusion

In conclusion, the presented findings contribute to the growing body of knowledge in the field of human–robot interaction, shedding light on the intricate dynamics of muscle coordination networks during the STS transition. The study opens avenues for further research and refinement of robotic assistance strategies to enhance the quality of life for individuals facing challenges in performing daily STS tasks.

## Data Availability

The raw data supporting the conclusions of this article will be made available by the authors, without undue reservation.
